# Genetic and chemical divergence among host races of a socially parasitic ant

**DOI:** 10.1002/ece3.4547

**Published:** 2018-11-06

**Authors:** Candice W. Torres, Maria A. Tonione, Santiago R. Ramírez, Joseph R. Sapp, Neil D. Tsutsui

**Affiliations:** ^1^ Department of Environmental Science, Policy, and Management University of California‐Berkeley Berkeley California; ^2^ Department of Evolution and Ecology University of California‐Davis Davis California; ^3^ Department of Ecology and Evolutionary Biology University of California‐Santa Cruz Santa Cruz California

**Keywords:** chemical ecology, cuticular hydrocarbons, genetic differentiation, host race, host–parasite relationship, speciation

## Abstract

Host–parasite associations facilitate the action of reciprocal selection and can drive rapid evolutionary change. When multiple host species are available to a single parasite, parallel specialization on different hosts may promote the action of diversifying natural selection and divergence via host race formation. Here, we examine a population of the kidnapper ant (*Polyergus mexicanus*) that is an obligate social parasite of three sympatric ant species: *Formica accreta*,* F. argentea*, and *F. subaenescens* (formerly *F. fusca*). Behavioral and ecological observations of *P. mexicanus* have shown that individual colonies parasitize only one species of host and that new *Polyergus* queens maintain host fidelity when establishing new colonies. To successfully adapt to a particular host, *Polyergus* ants may mimic or camouflage themselves with the species‐specific chemical cues (cuticular hydrocarbons) that their hosts use to ascertain colony membership. To investigate the extent of host specialization, we collected both genetic and chemical data from *P. mexicanus* that parasitize each of the three different *Formica* species in sympatry. We show that host‐associated genetic structure exists for both maternally inherited mitochondrial DNA data and biparentally inherited microsatellite markers. We also show that *P. mexicanus* can be distinguished by chemical profile according to host due to partial matching with their host. Our results support the hypothesis that host race formation is occurring among lineages of *P. mexicanus* that use different *Formica* hosts. Thus, this system may represent a promising model for illuminating the early steps of divergence, accumulation of reproductive isolation, and speciation.

## 1 INTRODUCTION

Divergent natural selection imposed by environmental gradients drives local adaptation, increases genetic and phenotypic differentiation, and can lead to reproductive isolation. A special case of adaptation leading to differentiation occurs when populations form host races, as when herbivores become adapted to different food plants (Forbes et al., 2017; Walsch, 1864) or when lineages of parasites become adapted to different hosts (Nosil, 2012; Nosil, Harmon, & Seehausen, 2009; Via, 2009). Under strong divergent natural selection, multiple host races may evolve, even when in sympatry (Criscione, Poulin, & Blouin, 2005; Filchak, Roethele, & Feder, 2000). However, even when host races evolve, complete speciation may not occur in the face of gene flow between the lineages that have specialized on different hosts (Feder, Chilcote, & Bush, 1988; Marchetti, Nakamura, & Gibbs, 1998; Spottiswoode, Stryjewski, Quader, Colebrook‐Robjent, & Sorenson, 2011).

Unlike conventional ecto‐parasites and endo‐parasites, social parasites take advantage of the brood care provided by the host species. Avian brood parasites, for example, exploit the rearing behavior of other birds by laying eggs in their hosts’ nests and provide convincing examples of host race formation (i.e., gentes, Gibbs et al., 2000; Marchetti et al., 1998) and even speciation in sympatry (Sorenson, Sefc, & Payne, 2003). Less well studied are the social parasites that exploit and manipulate entire colonies of ants, bees, wasps, or termites.

Social insect colonies provide ample opportunities for the evolution of cheating and parasitism. Thus, it is not surprising that at least 230 species of ants have evolved to be social parasites (Buschinger, 2009). One challenge of the parasitic lifestyle is the necessity for sufficiently abundant hosts (Foitzik & Herbers, 2001). The use of multiple different host species is one way to overcome this difficulty. However, host generalist species may experience lower efficacy when exploiting multiple hosts because traits that enable the parasite to use one host effectively may decrease its ability to use other hosts (Bauer, Bohm, Witte, & Foitzik, 2010; Guillem, Drijfhout, & Martin, 2014). For example, the precise colony‐mate recognition systems that have evolved in social insects and have been refined by long‐term coevolution between host and parasite lineages are a formidable barrier for some social parasites (Bonavita‐Cougourdan, Provost, Riviere, Bagneres, & Dusticier, 2004; D'Ettorre, Mondy, Lenoir, & Errard, 2002; Lenoir, D'Ettorre, Errard, & Hefetz, 2001). Thus, social parasites are likely to experience an evolutionary trade‐off between selection mediated by host scarcity (which should drive the evolution of generalist parasites) and selection mediated by host defenses (such as colony‐mate recognition), which should favor specialization by the parasite on a narrow range of hosts.

Kidnapper ants in the genus *Polyergus* (also known as Amazon ants, slave‐making ants, slave‐raiding ants, or pirate ants) are obligate social parasites that rely on their closely related *Formica* hosts to perform all colony tasks including brood care, nest maintenance, defense, and foraging (Topoff, Cover, & Jacobs, 1989; Trager, 2013). There appears to be high host fidelity from generation to generation because new *Polyergus* colonies are initiated when newly inseminated *Polyergus* queens accompany *Polyergus* workers on a raid of a nearby *Formica* colony (Topoff et al., 1989; Trager, 2013). Although this reproductive life history could promote maternal differentiation (e.g., in mtDNA), the extent of assortative mating, which could promote genomewide differentiation, remains unknown. The young *Polyergus* queen takes over the *Formica* colony by killing and replacing the resident *Formica* queen (Topoff, Cover, Greenberg, Goodloe, & Sherman, 1988). The *Formica* workers in this usurped colony then assist the new *Polyergus* queen in raising her first cohort of offspring. Eventually, these *Polyergus* workers begin to conduct raids on neighboring *Formica* colonies of the same species as the originally usurped colony, kidnapping *Formica* pupae, which then eclose in the *Polyergus* colony. This process replenishes the population of host workers in the *Polyergus* colony (Bono, Blatrix, Antolin, & Herbers, 2007; Topoff, LaMon, Goodloe, & Goldstein, 1985). Any single *Polyergus* colony only raids colonies of a single host species, even when other suitable host species are available (Bono et al., 2007; Goodloe, Sanwald, & Topoff, 1987; King & Trager, 2007), with the exception of the European *P. rufescens* (Trager, 2013). Therefore, there is likely to be a high degree of host fidelity from generation to generation within these lineages of *Polyergus*.

Kidnapper ants have evolved a number of adaptations to this obligate socially parasitic lifestyle, particularly in terms of chemical communication and colony recognition. For example, *Polyergus* queens accomplish colony takeover by acquiring the chemical cues of the killed host queen to gain acceptance by the resident *Formica* workers (Johnson, Vander Meer, & Lavine, 2001). Moreover, *Polyergus* workers may acquire some degree of chemical similarity with their hosts, by either synthesizing their own host colony recognition cues (chemical mimicry) or acquiring these cues from the hosts through chemical camouflage (Bonavita‐Cougourdan et al., 2004; D'Ettorre et al., 2002; Lenoir et al., 2001). This allows parasite and host to be integrated into a single colony and may also reduce opposition during host brood raids (Bagnéres and Lorenzi, 2010). In ants, the chemical cues for colony‐mate recognition are typically cuticular hydrocarbons (CHCs) (Bonavita‐Cougourdan, Clement, & Lange, 1987; Brandt, Wilgenburg, Sulc, Shea, & Tsutsui, 2009; Lahav, Soroker, Hefetz, & Vander Meer, 1999; Lalzar, Simon, Vander Meer, & Hefetz, 2010; Torres, Brandt, & Tsutsui, 2007). CHC chemical profiles are generally species‐specific (Emery & Tsutsui, 2016; Howard, 1993; Martin, Helanterä, & Drijfhout, 2008) and are often also colony‐specific (Brandt et al., 2009; Torres et al., 2007).

Here, we examine the molecular and chemical ecology of the socially parasitic kidnapper ant, *P. mexicanus* (formerly *P. breviceps*), which parasitizes three sympatric species of *Formica* at our study site: *F. accreta*,* F. argentea*, and *F. subaenescens*. Based on the apparent vertical transmission of host/parasite fidelity, we predict that the three lineages of *P. mexicanus* using these different hosts will exhibit patterns associated with host specialization, host race formation and, possibly, reproductive isolation. Specifically, we first test the hypothesis that lineages of *P. mexicanus* that use different host *Formica* will display significant genetic differentiation from each other. We test this hypothesis using both maternally inherited (mtDNA) and biparentally inherited (microsatellite) genetic markers. Next, we test the hypothesis that these *Polyergus* lineages also display significant phenotypic differentiation from each other in the pheromones used for colony recognition (cuticular hydrocarbons). We address this hypothesis by extracting cuticular hydrocarbons from field‐collected ants and analyzing them using gas chromatography–mass spectrometry (GC‐MS).

## 2 MATERIALS AND METHODS

### 2.1 Field site and collection information

**Figure 1:**
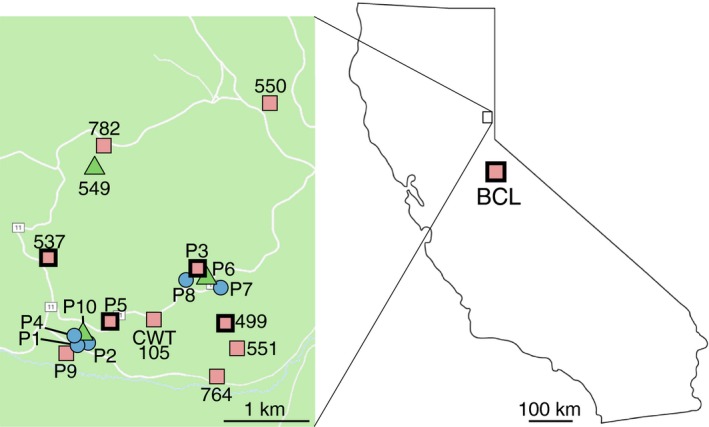
Map of sampling localities. Each symbol represents a sampled *Polyergus* colony. The color and shape of each symbol indicate the host *Formica* that is the host for each colony: *F. argentea* = blue circles, *F. accreta* = red squares, *F. subaenescens* = green triangles. *F. accreta* "A" colonies are shown with a bold border; *F. accreta* "B" colonies are shown with a hairline border

We conducted this study at Sagehen Creek Field Station, a University of California Natural Reserve located 13.5 km north of Truckee, CA (Figure 1). At this site, *P. mexicanus* parasitizes at least four different species of *Formica* in sympatry, including *F. subaenescens*,* F. argentea*,* F. accreta,* and, rarely, *F. neorufibarbis* (P.S. Ward, personal communication). During the summers of 2008, 2009, and 2010, we collected both *Polyergus mexicanus* workers and *Formica* host samples from 18 colonies (Supporting Information Appendix S1). For five of these colonies, the host colonies were identified as *F. argentea*, five were identified as *F. subaenescens* (formerly *F. fusca*), and eight were identified as *F. accreta* (but see Section 3). In addition, we included ants collected from a *P. mexicanus* colony collected at Blue Canyon Lake (BCL), Tuolumne Co., CA, located 130 km south of Sagehen Creek Field Station (Figure 1; Supporting Information Appendix S1). Host species were identified using the morphological key for the *Formica* fusca group developed by Francoeur (1973).

### 2.2 Genetic analysis

We extracted whole genomic DNA from the head or a single leg of *P. mexicanus* and *Formica* workers using the DNeasy Blood and Tissue Kit (Qiagen, Valencia, CA), following the recommended protocol and eluting the DNA in 200 μl of buffer AE. To determine whether *P*. *mexicanus* shows population structuring according to maternally inherited DNA, we amplified and sequenced two fragments of the mitochondrial gene, *cytochrome oxidase I* (COI) and *NADH dehydrogenase subunit 1* (NADH1), from one or two individuals of each genus (*Polyergus* and *Formica*) from each colony. To amplify COI for *P. mexicanus* individuals, we designed a forward primer, CI13Pbrev‐5′ CACTGCAATTTTACTTCTTT 3′, and paired it with a reverse primer designed for *P. samurai*, CI24 (Hasegawa, Tinaut, & Ruano, 2002). We amplified COI from all *Formica* using a primer we developed from the COI gene of *F. wheeleri*, COIFwhF –5′ TTCCTTTGCTTGTATGATCAATTT 3′, and paired it with CI24. To amplify NADH1, we used the primers from Liautard and Keller (2001): ND1a‐Fe‐F 5′ CTTTTAGAGATGCTATTAAATTGCTTA 3′ and ND1a‐Fe‐R 5′ TTGAATTAGATGATCATCCTATAAAAA 3′. Both mtDNA fragments were amplified in 12 µl PCR reactions which contained ~20 ng genomic DNA, 1× reaction buffer, 1.2 mM of MgCl_2_, 300 μM of each dNTP, 0.5 μM of each primer, and 0.04 units of Taq. The thermocycler conditions were as follows: a 3‐min initial denaturation of 94°C followed by 38 cycles of 45 s at 94°C, 45 s at 49°C and 1 min at 72°C, and ending with a 10 min extension step at 72°C. We verified the amplification of PCR products on 1% agarose gels and performed PCR cleanup using ExoSAP‐IT (USB). To sequence, we added approximately 33 ng of DNA from the purified PCR product, 0.46 pmol of primer, and 4 μl of BigDye Terminator v3.1 (ABI), and the following temperature program was used: 96°C for 1 min then 25 cycles of 96°C for 10 s, 50°C for 5 s, and 60°C for 4 min. PCR products were sequenced using a 96 capillary 3730xl DNA analyzer. We edited and aligned sequences using the program Geneious version 9.1.5 (Kearse et al., 2012). The final alignment including all *P. mexicanus* and *Formica* individuals and outgroup (specified below) was 648 bp of COI and 173 bp of NADH1.

We used the CIPRES Science Gateway (Miller et al., 2010) and MrBayes v 3.2.2 (Ronquist & Huelsenbeck, 2003) to build a single Bayesian majority rule consensus tree inferred from a combined total of 841 bp of COI and NADH1 from both *P. mexicanus* and *Formica spp*. hosts. We obtained *Camponotus chromaiodes* sequence from GenBank (COI: AY334392.1, NADH1: JX966368.1) as an outgroup to root the phylogeny. We determined the best fit nucleotide substitution model by Akaike information criterion (AIC) in PartitionFinder 1.1.1 (Lanfear, Calcott, Ho, & Guindon, 2012). The data were analyzed both partitioned by gene and by codon position. For the different codon positions in COI, we used the following models: GTR+I position 1, F81 position 2, GTR+G position 3. For the codon positions in NADH1, we used the following models: position 1 HKY+G, position 2, F81+I, position 3 HKY+G. When we partitioned by gene only, we used the GTR+I+G substitution model for COI and the K2P+G substitution model with NADH1. We also tested the GTR+G model, and the resulting tree was nearly identical to that recovered in the gene‐partitioned tree (below). We ran the analysis with default priors and settings for Markov chains for 3 × 10^7^ generations, sampled every 1,000 generations. As burn‐in, 25% of the trees were discarded, and convergence was evaluated by determining whether the average standard deviation of split frequencies fell below 0.01.

*Polyergus mexicanus* individuals from ten colonies (colonies P1‐P10) were also genotyped using six microsatellite primer pairs developed for *P. mexicanus* (pol1, pol2, pol3, pol4, pol5, and pol12, Bono et al., 2007) and two primer pairs developed for *F. yessensis* (Fy4 and Fy13, Hasegawa & Imai, 2004). We amplified each locus in 10 μl PCR reactions consisting of 1× reaction buffer, 1.25–2 mM of MgCl_2_, 300 µM of each dNTP, 0.8 µM of each primer, and 0.04 –0.075 units of GoTaq Flexi DNA Polyermase (Promega, San Luis Obispo, CA). For amplification, we used the following temperature program: initial denaturation for 5 min at 95˚C, 36 cycles of 30 s at 30˚C, 30 s at the appropriate annealing temperature, and a 30 s extension at 72˚C and ending with a final extension step for 5 min at 72˚C. Variations in reagent concentrations and annealing temperatures can be found in Supporting Information Appendix S2. We labeled all forward primers with fluorescent labels (VIC, 6FAM, PET, and NED) provided by Applied Biosystems (ABI; Carlsbad, CA, USA) and added LIZ size standard to the resulting PCR products. PCR products suspended in formamide were separated on an ABI 96 capillary 3730xl DNA Analyzer. We visualized and scored allele sizes using Peak Scanner v1.0 software (ABI).

Using the program MICROCHECKER v2.2.3 (Van Oosterhout, Hutchinson, Wills, & Shipley, 2004), we looked for evidence of possible scoring errors in microsatellite genotyping due to the presence of stutter, null alleles, and/or large allele dropout for all *P. mexicanus* individuals analyzed. We tested for deviations from Hardy–Weinberg equilibrium (HWE) and calculated allele frequency measurements and fixation indices (F) using GenAlEx v6.41 (Peakall & Smouse, 2006). The presence of linkage disequilibrium (LD) was detected using Arlequin v 3.1 (Excoffier, Laval, & Schneider, 2005).

We genotyped 193 individual *P. mexicanus* (59 *P. mexicanus* with *F. accreta* from three colonies, 95 *P*. *mexicanus* with *F*. *argentea* from five colonies, 39 *P*. *mexicanus* with *F*. *subaenescens* from two colonies). Results from MICROCHECKER revealed that, while there was no sign of large allele dropout, five out of the eight loci were flagged as possibly having null alleles. Taking all *P. mexicanus* as a single population, only one out of the eight loci (Fy4) was in HWE. When testing for HWE at the levels of colony and host, we found no loci consistently in equilibrium, likely due to population fragmentation across colonies and the local scale of population sampling in this study.

For the entire population of *P. mexicanus*, the average number of alleles per locus per colony was 2.56 ± 0.48 (*SD*), and the average expected heterozygosity was 0.39 ± 0.08. The average number of private alleles per colony was 0.20 ± 0.21. The average fixation index (*F*) across all loci was 0.181 ± 0.16. When analyzing *P. mexicanus* using the species of host as a population unit, the average fixation indices per locus were as follows: 0.023 ± 0.34 (*F. accreta*), 0.027 ± 0.21 (*F. argentea*), and 0.060 ± 0.34 (*F. subaenescens*). We found all loci were linked across all possible locus pairings when considering all sampled *P. mexicanus* as one population. Therefore, we designated populations according to host and then viewed occurrence of LD across all possible locus pairs to see whether LD in the whole population may be a result of population structuring according to host. *P. mexicanus* parasitizing *F. accreta* showed 95%, *P. mexicanus* parasitizing *F. argentea* showed 100%, and *P. mexicanus* parasitizing *F. subaenescens* showed 86% LD. Like tests for HWE, LD is also sensitive to demographic events, particularly coancestry (Kaeuffer, Reale, Coltman, & Pontier, 2007), which is likely within our population.

To determine whether *P. mexicanus* colonies are distinguishable genetically according to host, we performed two types of analysis on microsatellite data that examine population clustering: one in the program STRUCTURE v2.3.3 (Falush, Stephens, & Pritchard, 2003) and the other a discriminate analysis of principal components (DAPC) in the program R. In STRUCTURE, we explored a range of possible numbers of population clusters (*K*) from 2 to 10 (the total number of colonies sampled) using a burn‐in length of 50,000 followed by 100,000 Markov chain Monte Carlo (MCMC) repetitions. We used both the *admixture* and the *correlated allele frequency* models under default settings. STRUCTURE has two options that allow the user to set up prior populations of origin using “popflag” and “popinfo.” During initial runs with and without these options, we determined there were no detectable differences between them. Here, we present results from runs with both population options turned on and the species of host parasitized by the genotyped *P. mexicanus* individual set as prior “populations.” Since we were particularly interested in whether *P. mexicanus* at our site were genetically grouped according to host, we performed an additional 10 runs each of *K* = 3 (for the three species of hosts identified morphologically) and *K* = 4 (to account for a possible additional cryptic host indicated in the mtDNA results, see below) at 100,000 burn‐in length and 1,000,000 MCMC repetitions. We summarized the clustering patterns found in our runs of *K* = 3 and *K* = 4 using CLUMPP v1.1.2 (Jakobsson & Rosenberg, 2007) and visualized them using DISTRUCT v1.1 (Rosenberg, 2004).

If the assumptions of STRUCTURE are not met (i.e., departures from HWE and LD are not associated with population structure but instead with inbreeding or scoring errors), STRUCTURE may oversplit a population (Pritchard et al., 2010). Therefore, we also performed a DAPC analysis in the program R using the function *adegenet* 1.3–4 (Jombart, 2012). DAPC is a multivariate analysis method that combines the advantages of principal components analysis (PCA) and discriminant analysis (DA) to determine assignment of individuals to genetic clusters (Jombart, Devillard, & Balloux, 2010). Because this method transforms genetic data using PCA, the assumptions of HWE and no LD do not need to be met to explore genetic clustering. DAPC requires groups be defined prior to conducting the analysis and biologically defined groups are recommended as the most useful way of examining group membership (Jombart, 2012). As such, clusters were defined according to species of host *P. mexicanus* workers were found with before implementing the function *dapc* in the *adegenet* package.

### 2.3 Chemical data collection and analysis

We analyzed cuticular hydrocarbon profiles from 18 to 20 individual *Polyergus* and *Formica* hosts from each of 19 colonies. After collecting *P. mexicanus* and *Formica* workers from the field, we freeze‐killed the specimens and soaked each individual for 10 min in 200 μl of chromatography grade hexanes in 9 mm glass GC vials (Agilent Technologies, Santa Clara, CA). Ants were then removed from the solvent, allowed to air‐dry, and were transferred into 95% ethanol for genetic analysis (see previous section). The hexane extracts were stored at −20°C and kept on dry ice for transport back to the laboratory. We evaporated the hexane from each sample under nitrogen, re‐eluted the extract in 40 μl of hexane, and transferred it to a small glass insert with polymer spring (Varian, Palo Alto, CA).

To analyze the cuticular hydrocarbon (CHC) extracts, 2 μl was injected in splitless mode into an Agilent 7890A Gas Chromatograph (GC) coupled with a 5975C Mass Spectrometer (MS) with triple axis detector. We used an Agilent DB‐5, 30 m × 320 µm × 0.24 µm capillary column to separate the components of individual CHC profiles using the following temperature program with a 5‐min solvent delay: 70°C for 2 min, 30°C/min to 200°C, and then 3°C/min to 325°C for 10 min. The MS was set to scan from 40 to 600 amu.

We viewed and analyzed the components of all chemical profiles using the software MSD ChemStation (v.E.02.00.493, Agilent Technologies, Santa Clara, CA). From each of the 10 colonies, we sampled two or more chromatograms from each genus (*Polyergus* and *Formica*) that had the highest concentration of total chemical peaks to custom‐build a library of chemical components for both genera. With this library, we assessed the presence or absence of 105 chemical peaks in the CHC profiles of all individuals sampled by creating a library search report in ChemStation. This procedure allowed us to match the mass spectrum of each chemical peak analyzed with a mass spectrum from the library we customized. We used default settings for the library match options with the following exceptions: U + A (4), Flag (1), Min Est Purity (50). We used ChemStation integrator and the “autointegrate” function to detect and calculate the amounts of each chemical peak present in the profile in units of peak area, and only peaks that matched with one of the 105 library entries were counted as present. Relative amounts of each chemical peak were determined by calculating the percent area contribution of library‐matched peaks identified by the ChemStation integrator on a per individual basis.

We performed a nonmetric multidimensional scaling (NMDS) analysis on the relative peak areas of *P. mexicanus* profiles using the package *vegan* and the function *metaMDS* in the program R (https://cran.r-project.org, v2.14.0). Chemical peaks that were not present or that could not be detected by the ChemStation integrator were counted as having an area of zero. We used the percent peak area to calculate a Bray–Curtis pairwise distance matrix and performed an analysis of similarity (ANOSIM) on this matrix to test for possible differences in the chemical profiles according to host using the function *anosim* in R. Next, we combined the chemical data from *P*. *mexicanus* with the data of their *Formica* hosts into a single NMDS analysis to see whether *P. mexicanus* workers clustered more closely with their resident host than with other available host species.

In total, we analyzed the relative abundance of 105 chemical peaks across all *P. mexicanus* and *Formica* individuals. To determine which of these 105 peaks most likely contributed to any separation of *P. mexicanus* by hosts, we performed a SIMPER (similarity percentage) analysis using PRIMER 6 (Clarke, 1993), which is based on decomposition of the Bray–Curtis dissimilarity index.

## 3 RESULTS

### 3.1 Genetic analysis

#### 3.1.1 Mitochondrial sequence data

**Figure 2:**
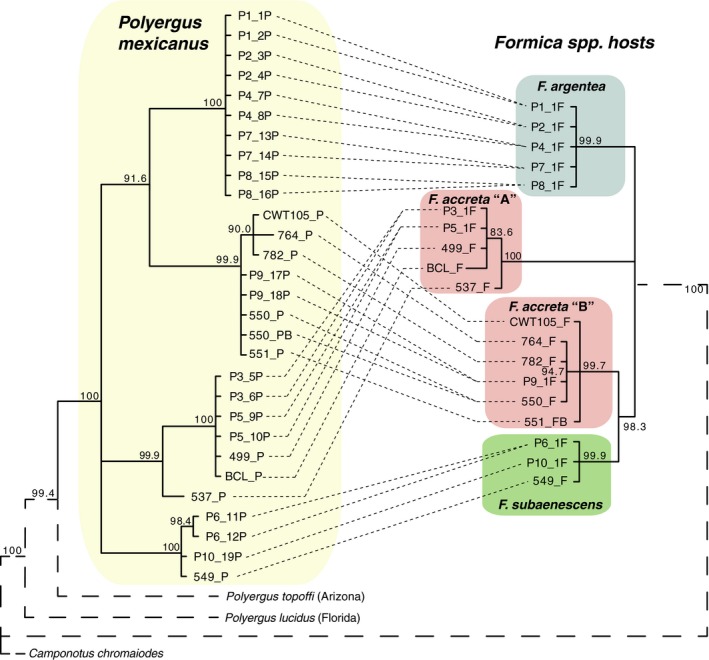
Mitochondrial phylogeny of both parasite (*Polyergus mexicanus*) and host (*Formica* spp.). Topology of the tree has been drawn so *P. mexicanus* can be compared to their *Formica* hosts. Fine dashed lines between tips connect parasites and hosts from the same colony. Node values represent Bayesian posterior probability values. Branches shown as dashed lines are not drawn to scale

The host (*Formica*) mitochondrial tree resolved four major monophyletic groups rather than three that matched the nominal species (Figure 2, right). Two of the host species, *F. argentea* and *F. subaenescens*, were recovered as monophyletic groups. However, our samples of the third host species (*F. accreta*) were divided between two separate, well‐supported clades. In the codon‐partitioned tree, one of these (*F. accreta* “B”) was placed as sister to *F. subaenescens*, whereas the other (*F. accreta* “A”) was recovered as a clade with an unresolved relationship relative to *F. argentea* and *F. accreta* “B” + *F. subaenescens* (Figure 2, right). These same four groups were recovered in the gene‐partitioned tree, but with *F. accreta* “A” placed as sister to the other three clades. Notably, two of the *Formica* specimens that fell within the *F. accreta* “A” (499_F and 537_F) were initially identified as *F. subaenescens* based on morphology.

The *Formica* sample from Blue Canyon Lake (“BCL_F” in Figure 2), which was collected ~130 km south of Sagehen Creek Field Station, was placed within *F. accreta* “A.” Based on morphology, this specimen was initially identified as *F. cf. argentea* (P.S. Ward, pers. comm.).

Similar to the pattern observed for the *Formica* hosts, our reconstruction of the intraspecific relationships among *Polyergus* parasites yielded four well‐supported mitochondrial lineages (Figure 2, left). Remarkably, these *Polyergus* clades perfectly matched the four observed *Formica* host clades, although the relationships among the four *Polyergus* clades were somewhat different. Interestingly, this pattern even held for the 130 km‐distant BCL colony despite its geographic distance from all other Sagehen *Polyergus*: the *Polyergus* enslaving this *Formica* also fell with the corresponding clade of *F. accreta* “A”‐enslaving *Polyergus* (Figure 2, left).

#### 3.1.2 Microsatellite data

**Figure 3:**
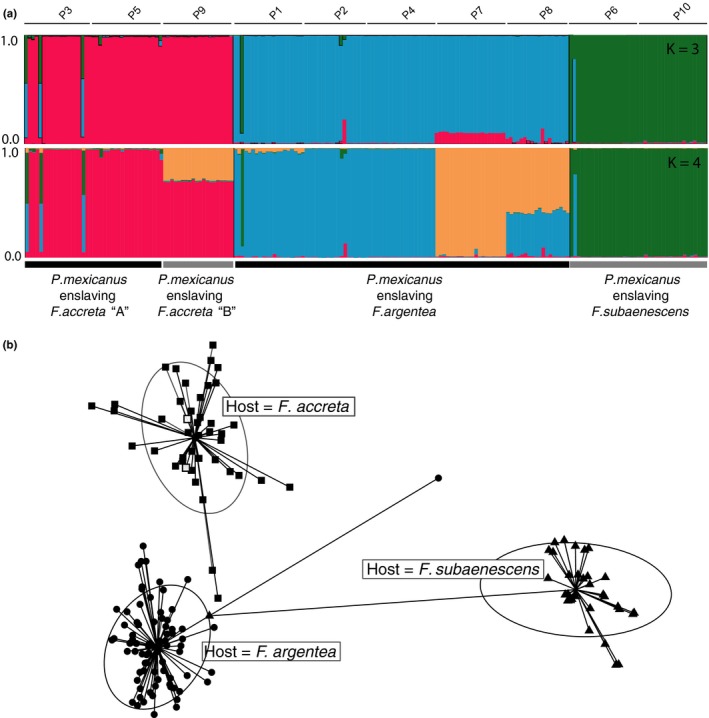
Genetic analysis of microsatellite data from *Polyergus mexicanus* parasitizing one of three *Formica* hosts. (a) Results from the STRUCTURE analysis at *K* = 3 and *K* = 4. Alpha‐numeric codes across the top indicate colony identity. (b) DAPC plot of *P. mexicanus* individual genotypes based on microsatellite data. Individuals are plotted according to their host: *F. accreta* “A” (filled squares), *F. accreta* “B” (open squares), *F. argentea* (circles), and *F. subaenescens* (triangles). Large circles drawn around points represent inertia ellipses that graphically summarize the cloud of points

The STRUCTURE analysis of *P. mexicanus* revealed an overall pattern of structuring that matched the morphological host species identification (at *K* = 3, Figure 3a). The division of *Polyergus* using *F. accreta* “A” and “B” as host that was seen in the *Polyergus* mitochondrial tree (Figure 2, left) was not evident at microsatellite loci (Figure 3a). Specifically, colonies P3 and P5 (host: *F. accreta* “A”) were not distinguishable from colony P9 (host: *F. accreta* “B”). At *K* = 4, there was no biologically relevant fourth cluster with the exception of colony P7 (host: *F. argentea*), which was fully assigned to this cluster (Figure 3a; orange).

Likewise, the DAPC plot showed a pattern of clustering of *P. mexicanus* according to host species (Figure 3b). Percent assignments of *P. mexicanus* correctly grouped by host as calculated by the DAPC were as follows: 92.3% for *F*. *accreta*, 98.9% for *F. argentea*, and 98.3% for *F. subaenescens*. As in the STRUCTURE analysis (but unlike in the mtDNA tree), the *Polyergus* from colony P9 (host:*F. accreta* “B”) grouped with all others enslaving *F. accreta,* with the mitochondrial division of *F. accreta* not evident.

### 3.2 Chemical analysis

**Figure 4:**
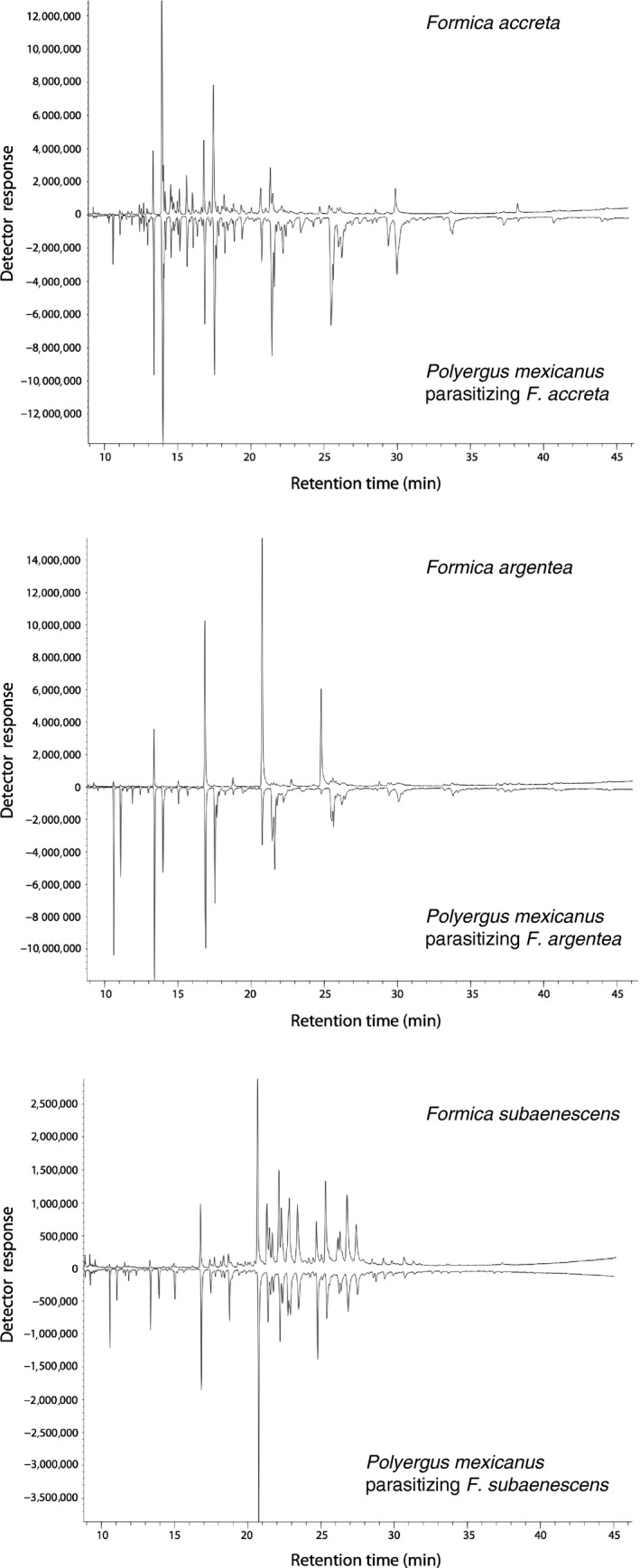
Representative chemical profiles by GC‐MS from one *Polyergus mexicanus* (bottom of each chromatogram) and its respective host species from the same colony (top of each chromatogram)

**Figure 5:**
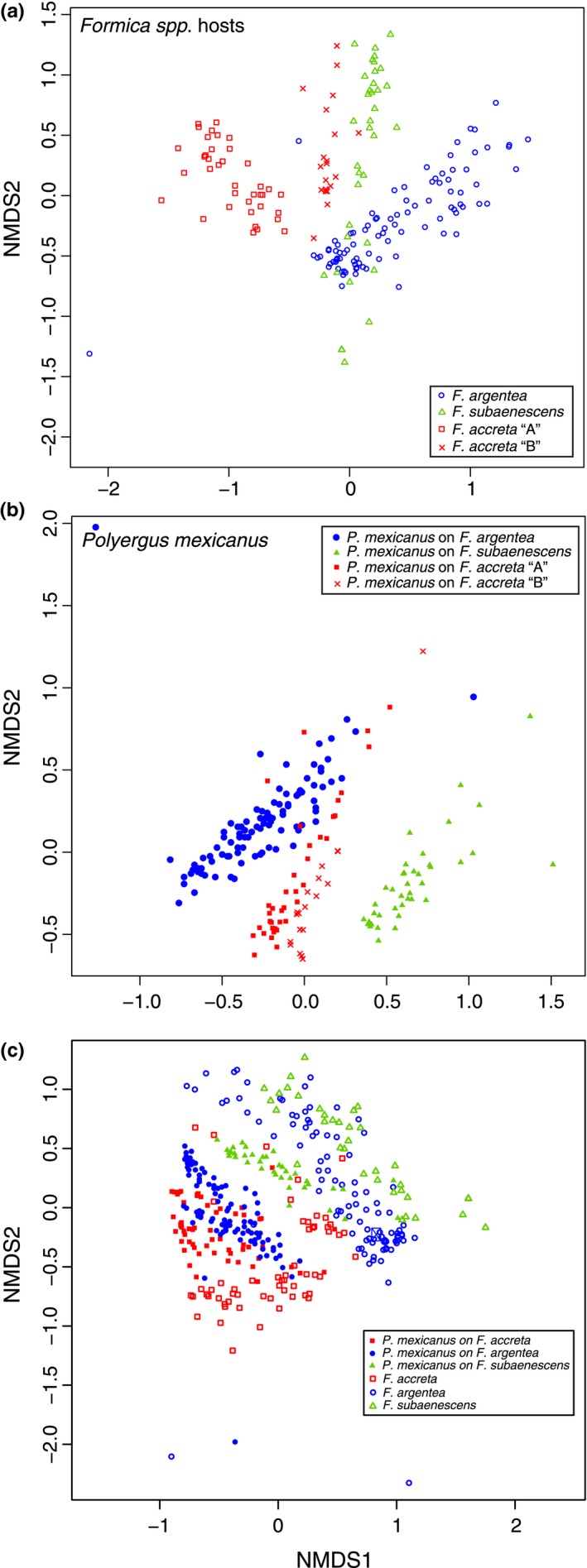
NMDS of chemical profiles of (a) enslaved *Formica*, (b) *P. mexicanus* social parasites, and (c) both host and parasite together. Each symbol represents an individual ant; *Formica* host species and *Polyergus* host races coded by color. Hosts and parasites from colony “P9” indicated by red “x” symbols

**Figure 6:**
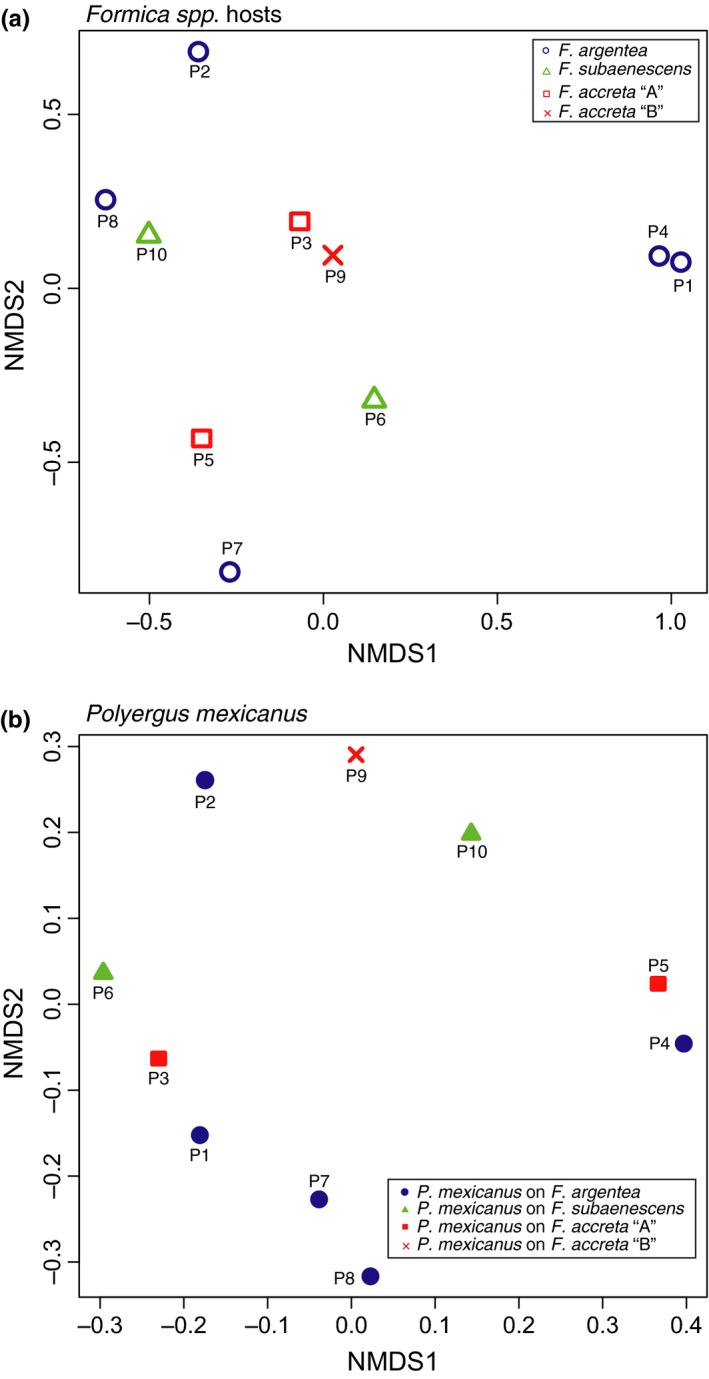
NMDS plots of chemical profiles by colony. (a) Host *Formica* workers from *P. mexicanus* colonies. (b) *P. mexicanus* workers from the same colonies

Qualitatively, the chemical profiles of the three species of *Formica* parasitized by *P. mexicanus* showed species‐specific differences (Figure 4, upper chromatograms). Taken as a whole, across all individuals, ANOSIM and NMDS analyses of these parasitized *Formica* confirmed chemical grouping according to morphologically defined species (Figure 5a; ANOSIM results: *R* = 0.554, *p* = 0.001). One exception was *F. accreta* “B” colony P9 that appeared more similar to *F. subaenescens* than to *F. accreta* “A” (Figure 5a, red x symbols), a relationship reminiscent of the mtDNA relationships (Figure 2; right). There was no significant chemical differentiation when the analysis was performed at the colony level (Figure 6). This may be due, in part, to the fact that *Formica* from each *Polyergus* colony actually originate from a variety of different natal colonies, thus leading to high diversity among individuals from a single colony's enslaved host population.

There also appeared to be qualitative differences among the chemical profiles of *P. mexicanus* parasitizing the three different species of *Formica* (Figure 4, bottom chromatograms). The NMDS plot of all individual *P. mexicanus* profiles showed that *P. mexicanus* individuals largely clustered into one of three groups according to the host species (Figure 5b), and the results from the ANOSIM analysis of individual chemical profiles confirmed these host‐specific differences to be significant (*R* = 0.678, *p* = 0.001). Colony‐level ANOSIM revealed significantly lower CHC diversity within versus among colonies (*R* = 0.876; *p* = 0.001), but there was no clear association among colonies that used the same host species (Figure 6).

Interestingly, and unlike the pattern seen in the *Formica* host, the *P. mexicanus* workers from colony P9 (host: *F. accreta* “B”) possessed chemical profiles that were most similar to the rest of the *Polyergus* enslaving *F. accreta* “A” (red x symbols in Figure 5b). When all parasitized *Formica* were included with their parasites in a single NMDS analysis, *P. mexicanus* individuals tended to group with their host species, with the exception of *P. mexicanus* parasitizing *F. argentea* (Figure 5c).

The SIMPER analysis revealed that 15 of the 105 peaks analyzed by GC/MS contributed to over 50% of the chemical differences found between *F. accreta* and *F. argentea* (Supporting Information Appendix S3). Twelve out of the 105 peaks analyzed distinguished *F. subaenescens* and *F. accreta* by over 50%. *Formica subaenescens* and *F. argentea* had 11 of 105 peaks that differentiated them by over 50%. Five of these distinguishing peaks were found across all three comparisons mentioned above. Overall, these CHCs were either linear alkanes or branched alkanes with one, two, or three methyl groups, ranging from 21 to 33 carbons in length (Supporting Information Appendix S3).

## 4 DISCUSSION

### 4.1 Overview

In contrast to null expectation of panmixia, we found extensive genetic differentiation among sympatric colonies of *P. mexicanus* social parasites, at both mitochondrial loci and nuclear microsatellites. Remarkably, this differentiation matched host use, suggesting that *Polyergus* host races have evolved to specialize on different host *Formica*. Consistent with this, samples from a geographically distant site grouped with the appropriate host race, rather than sister to all Sagehen Creek samples, as would be expected under a typical pattern of genetic isolation by distance. However, different patterns were evident between the mtDNA and nuclear microsatellites, suggesting different patterns of maternal versus paternal gene flow or different rates of molecular evolution. Analysis of chemical phenotypes that are crucial for ant social behaviors (cuticular hydrocarbon pheromones; CHCs) produced mixed results. When all individuals were included separately, *Polyergus* colonies that used different *Formica* hosts were clearly differentiated. However, these differences were not observed when the analysis was performed with individuals grouped into colonies.

Overall, these data, combined with the known maternal vertical transmission of host species identity during colony founding, suggest that differentiation among lineages that use different hosts has led to the evolution of sympatric *Polyergus* host races. In addition, differentiation of *P. mexicanus* with respect to host species was observed at the nuclear microsatellites, suggesting that assortative mating may also be occurring. Interestingly, these host races of *P. mexicanus* are cryptic lineages, showing no obvious morphologically distinguishable characteristics (J.C. Trager, personal communication). In future studies, it would be useful to assess the extent of reproductive isolation among these lineages and determine whether they represent the formation of incipient species in sympatry.

### 4.2 Genetic differentiation among host races

The genetic differentiation that we found among *Polyergus* lineages is consistent with the evolution of host specificity and high host fidelity, leading to reduced gene flow among parasites that use different hosts (Archie & Ezenwa, 2011; Criscione et al., 2005). The difference that we observed between mtDNA and microsatellites with respect to the *F. accreta* host race may indicate different patterns of male versus female gene flow. The natural history of *Polyergus* colony founding suggests a mechanism for this difference: New queens disperse locally, on foot, into colonies of the same host species as their natal colony, whereas males fly in from more distant surrounding colonies that potentially use a different host species. Although the pattern of differentiation was not identical between mtDNA and microsatellites, the clear nuclear differentiation among *Polyergus* using different *Formica* hosts indicates that male‐mediated gene flow is not occurring randomly. Instead, it appears that some additional form of reproductive isolation, such as assortative mating, is also occurring. The exact dynamic of male versus female gene flow across host species remains unknown, but is likely to be an important force in determining the extent of host specialization.

An additional mechanism that might further limit gene flow is assortative mating between kidnapper ant sexuals, as *P. mexicanus* queens and/or males might preferentially select mates from colonies that parasitize the same *Formica* species as their natal nest. In *P. mexicanus*, queens disperse on foot and release pheromones from their mandibular glands to attract males for mating (Greenberg et al., 2007; Greenberg, Aliabadi, McElfresh, Topoff, & Millar, 2004). The specificity of this queen sex pheromone is unknown, and little is known about the details of mating behavior in *P. mexicanus*.

Cuticular hydrocarbons may also play an important role in mate choice (Howard, Jackson, Banse, & Blows, 2003). For example, a study by Beibl, Buschinger, Foitzik, and Heinze (2007) found that sexuals of the socially parasitic ant, *Chalepoxenus mullerianus*, obtain some of their CHCs from their host workers. This type of “gestalt” model for CHC‐sharing among nestmates is well known in other ant species (Crozier & Dix, 1979; Soroker, Vienne, & Hefetz, 1995). In this way, in *Polyergus*, new virgin queens could acquire CHCs from *Formica* hosts in their natal nests. This could then produce reproductive isolation among *Polyergus* host races if (a) these queens are disproportionately attractive to males from the same host race, (b) these queens are more effective at infiltrating nests of their host species during colony founding, or (c) this CHC‐sharing process produces a behavioral preference in dispersing queens for their natal host species. When coupled with the known reproductive life history (gynes disperse with raiding nestmate workers), these processes could further reinforce host race fidelity. Indeed, Beibl et al. (2007) showed that male *Chalepoxenus* ants showed more interest and engaged in more mating activities with queens reared on the same host species. Examining the chemical profiles of *P. mexicanus* sexuals and performing choice experiments between *P. mexicanus* queens and males reared from different species of host might allow us to better assess whether CHCs play a role in assortative mating. Similarly, manipulative experiments in which CHCs are transferred between reproductives from different host races could be a powerful approach for testing the potential roles of these pheromones in reproductive isolation.

Another potential (but not mutually exclusive) driver of differentiation could be postzygotic selection against “hybrid” kidnapper ant colonies produced from the matings of sexuals originating from nests that parasitized two different hosts. For example, worker offspring of such crosses might be less effective at raiding the maternal host species due to reduced CHC matching or the expression of aberrant behaviors during raids. Although we did not discover any apparent hybrids in this study, more extensive sampling might reveal such colonies.

### 4.3 Chemical adaptation

The total dependence of *P. mexicanus* on *Formica* likely imposes selection on the parasite to elude the recognition system of its hosts. Mimicry of host recognition cues, such as CHCs, is a common adaptive strategy used by such social parasites (Bagnéres and Lorenzi, 2010). Because CHC cues are often species‐specific (Emery & Tsutsui, 2016; Howard, 1993; Martin, Helantera, Kiss, Lee, & Drijfhout, 2009), we predicted that *P. mexicanus* specializing on a particular host species might have chemical profiles matching their host species and that this would result in *P. mexicanus* colonies becoming distinct from one another according to host species. We observed patterns consistent with this when all individuals were analyzed together without taking into account their colony of origin. Chemical profiles of *P. mexicanus* workers generally clustered together to form three groups corresponding to one of the three species of host they parasitize. However, this pattern was not evident in our analysis at the colony level. Although odor sharing between host and parasite may account for some of this grouping, the distinctly different patterns displayed by the *F. accreta* “B” host versus the *Polyergus* that parasitized it (red x symbols in Figure 5a vs. b) show that decoupling can occur between the odor profiles of cohabitating host and parasite.

To further examine the extent to which *P. mexicanus* may be chemically adapted to a particular species of *Formica*, we plotted the cuticular profiles of both parasites and their hosts together (Figure 5c). We found that, although *P. mexicanus* parasitizing *F. accreta* and *F. subaenescens* clustered closely to their respective hosts, *P. mexicanus* parasitizing *F. argentea* did not cluster as closely to their hosts. This suggests that *P. mexicanus* enslaving *F. argentea* may not be as chemically well matched to their host as *P. mexicanus* parasitizing the other two *Formica* species or that less odor sharing occurs among nestmates within these colonies. Still, we cannot be certain whether this relatively lower level of matching decreases the efficacy of *P. mexicanus* enslaving *F. argentea* relative to *P. mexicanus* enslaving other hosts. Moreover, it remains unknown to what extent individual CHCs contribute to the relevant recognition behaviors. Further observations of *P. mexicanus* raiding behavior and targeted chemical analyses are needed to address these questions. Additionally, *P. mexicanus* still appears to maintain its own genus‐specific profile, and there are likely some limitations to how closely *P. mexicanus* can match their host profile, particularly if they were adapted to a different host species sometime in the past.

Previous studies have shown that social parasites often have CHC profiles that match their host, but the degree of matching may vary (Bagnéres and Lorenzi, 2010). The extent of chemical adaptation by social parasites may be driven by factors such as the local availability of hosts, their ecological interactions with such hosts, and ability to obtain or synthesize chemicals that match their host. A few studies have examined how the availability of multiple hosts in sympatry may affect the chemical adaptation of social parasites. In one study, Brandt, Heinze, Schmitt, and Foitzik (2005) found that the socially parasitic ant, *Protomognathus americanu*s, had chemical profiles that appeared to be intermediate between sympatrically distributed host species in the genus *Temnothorax*. However, in another location where only one species of host occurred, the parasite had a chemical profile that more closely matched its host. The authors concluded that, at the site with two sympatric hosts, *Protomognathus* acted more as a generalist than a specialist. In another study, Bauer et al. (2010) found that the parasite, *Harpagoxenus sublaevis*, more closely matched one of two host species of *Leptothorax*. However, unlike *P. mexicanus*,* H. sublaevis* can parasitize both species of *Leptothorax* within the same colony. In these mixed host colonies, the chemical profile of *Harpagoxenus* more closely resembled one of the two host species. Nash, Als, Maile, Jones, and Boomsma (2008) found that the chemical profiles of the socially parasitic caterpillars of *Maculinea alcon* did not display host specificity when two species of *Myrmica* were available at the same site at equal frequencies. Instead, their profile appeared to be a mixture of cues from both hosts. In contrast, close matching of the *Myrmica* host profiles was apparent at two other sites where only one host was primarily used by the parasitic caterpillar.

Although chemical profiles of *P. mexicanus* workers enslaving *F. argentea* do not seem to match their host as closely as the *Polyergus* parasitizing *F. accreta* “A” and *F. subaenescens*, chemical profiles of *P. mexicanus* colonies at our site do generally appear to be distinguishable according to host, thus suggesting some level of host specificity. This is in contrast to two of the studies mentioned above (Brandt et al., 2005; Nash et al., 2008), where chemical specialization and separation by host species occurring in sympatry was not apparent. Further studies that examine the cuticular chemical profiles of free‐living *Formica* from our field site and nearby populations of *P. mexicanus* that parasitize only one host would help clarify the extent of this chemical adaptation.

### 4.4 *Formica accreta* “B”

Interestingly, both hosts and parasites from the *F. accreta* “B” clade displayed conflicting patterns. The hosts in this colony, morphologically identified as *F. accreta*, were more similar to *F. subaenescens* in mtDNA sequence and CHC phenotype. Further study may reveal that this lineage is a cryptic, undescribed species of *Formica*. Similarly, the *P. mexicanus* that parasitize *F. accreta* “B” formed a distinct and well‐supported monophyletic group based on mtDNA but, at nuclear loci, grouped with the other *Polyergus* that used *F. accreta* as host. It is difficult to determine exactly how this lineage originated. One possibility is that different patterns of maternal versus paternal gene flow have produced different patterns at nuclear and mitochondrial loci. Future studies may resolve this mystery by sampling from a broader geographic area and applying more extensive genetic and genomic analyses.

### 4.5 Host race formation and incipient speciation

At present, we cannot determine whether the patterns observed here represent the occurrence of cryptic species that occur in sympatry (secondary contact) or sympatric speciation. Providing clear geographic and population genetic evidence for speciation and host race formation in sympatry is challenging (Fitzpatrick, Fordyce, & Gavrilets, 2008; Via, 2001). Support for host race formation in ant social parasites has not been previously reported and has been rarely examined (but see Fanelli, Henshaw, & Cervo, 2005). Because our genetic data indicate restricted biparental gene flow among the three *P. mexicanus* host races within a small geographic area, a tantalizing conclusion is that *P. mexicanus* at our study site is forming host races and undergoing the first steps of speciation in sympatry. However, it is also possible that the overlap of these three host races at our field site represents secondary contact among lineages that diverged elsewhere. Examination of populations across a larger geographic area and other possible host associations will clarify the evolutionary pathways that led to this distribution of sympatric host races.

Strong divergent selection on a particular trait may jumpstart the speciation process and result in reduced gene flow between subpopulations (Nosil et al., 2009). Although our results clearly reveal patterns of genetic divergence consistent with this differentiation, the phenotypic traits driving this process are not yet clear. Host race formation may be viewed as an early stage in the process of speciation, with incomplete genetic isolation and development of complete reproductive barriers not yet fully established (Drès & Mallet, 2002). Studies that focus on these early stages of differentiation are crucially important for understanding the speciation process, in part because differences that arise later become confounded with the actual early drivers of differentiation (Via, 2009). Thus, studies such as ours serve as important starting points for increasing our understanding of the conditions by which new species form.

## AUTHOR CONTRIBUTIONS

CWT designed the experiment, collected the data in the field and the laboratory, and performed data analysis. MAT collected and analyzed mtDNA sequence data. SRR assisted with data analysis. JRS performed fieldwork and contributed samples. NDT contributed to experimental design and assisted with data analysis. CWT and NDT designed figures and wrote the manuscript.

## DATA ACCESSIBILITY

Voucher specimens (thorax and abdomen) deposited in the Bohart Museum of Entomology, UC Davis. DNA sequences: GenBank Accession numbers MH633506–MH633555, MH645523–MH645572.
