# Supplementary material for: Genetic and chemical divergence among host races of a socially parasitic ant
Source: Ecol Evol. 2018 Nov 6;8(23):11385–98. doi: 10.1002/ece3.4547 (PMC6303767; doi:10.1002/ece3.4547)
Supplement: Supplementary file 1 [file ECE3-8-11385-s001.pdf]

Appendix 1. Specimen information.

Specimen ID	colony	genus	species	collection locale	county	state	collector	latitude	longitude
CWT068_1P	CWT068	*Polyergus*	*topoffi*	Portal	Cochise	Arizona	CW Torres	31.909633	-109.148483
CWT105_1P	CWT105	*Polyergus*	*mexicanus*	Sagehen	Nevada	California	CW Torres	39.438333	-120.252222
CWT105_1F	CWT105	*Formica*	*accreta* "B"	Sagehen	Nevada	California	CW Torres	39.438333	-120.252222
For499	499	*Formica*	*accreta* "A"	Sagehen	Nevada	California	JR Simms	39.436918	-120.242106
For537	537	*Formica*	*accreta* "A"	Sagehen	Nevada	California	JR Simms	39.443494	-120.264795
For549	549	*Formica*	*subaenescens*	Sagehen	Nevada	California	SA Cooper	39.452363	-120.256382
For550	550	*Formica*	*accreta* "B"	Sagehen	Nevada	California	JR Sapp	39.458085	-120.236496
For551B	551	*Formica*	*accreta* "B"	Sagehen	Nevada	California	JR Sapp	39.434619	-120.2411
For764	764	*Formica*	*accreta* "B"	Sagehen	Nevada	California	JR Sapp	39.432398	-120.243866
For782	782	*Formica*	*accreta* "B"	Sagehen	Nevada	California	JR Sapp	39.454345	-120.255692
JRK1001_1P		*Polyergus*	*lucidus*		Leon	Florida	JR King	30.279895	-84.340082
P1_1F	P1	*Formica*	*argentea*	Sagehen	Nevada	California	CW Torres	39.436083	-120.261194
P1_1P	P1	*Polyergus*	*mexicanus*	Sagehen	Nevada	California	CW Torres	39.436083	-120.261194
P1_2P	P1	*Polyergus*	*mexicanus*	Sagehen	Nevada	California	CW Torres	39.436083	-120.261194
P10_19P	P10	*Polyergus*	*mexicanus*	Sagehen	Nevada	California	CW Torres	39.43625	-120.260611
P10_1F	P10	*Formica*	*subaenescens*	Sagehen	Nevada	California	CW Torres	39.43625	-120.260611
P2_1F	P2	*Formica*	*argentea*	Sagehen	Nevada	California	CW Torres	39.435778	-120.26075
P2_3P	P2	*Polyergus*	*mexicanus*	Sagehen	Nevada	California	CW Torres	39.435778	-120.26075
P2_4P	P2	*Polyergus*	*mexicanus*	Sagehen	Nevada	California	CW Torres	39.435778	-120.26075
P3_1F	P3	*Formica*	*accreta* "A"	Sagehen	Nevada	California	CW Torres	39.441778	-120.245778
P3_5P	P3	*Polyergus*	*mexicanus*	Sagehen	Nevada	California	CW Torres	39.441778	-120.245778
P3_6P	P3	*Polyergus*	*mexicanus*	Sagehen	Nevada	California	CW Torres	39.441778	-120.245778
P4_1F	P4	*Formica*	*argentea*	Sagehen	Nevada	California	CW Torres	39.436444	-120.261000
P4_7P	P4	*Polyergus*	*mexicanus*	Sagehen	Nevada	California	CW Torres	39.436444	-120.261000
P4_8P	P4	*Polyergus*	*mexicanus*	Sagehen	Nevada	California	CW Torres	39.436444	-120.261000
P5_10P	P5	*Polyergus*	*mexicanus*	Sagehen	Nevada	California	CW Torres	39.437472	-120.256667
P5_1F	P5	*Formica*	*accreta "A"*	Sagehen	Nevada	California	CW Torres	39.437472	-120.256667
P5_9P	P5	*Polyergus*	*mexicanus*	Sagehen	Nevada	California	CW Torres	39.437472	-120.256667

P6_11P	P6	*Polyergus*	*mexicanus*	Sagehen	Nevada	California	CW Torres	39.441583	-120.245639
P6_12P	P6	*Polyergus*	*mexicanus*	Sagehen	Nevada	California	CW Torres	39.441583	-120.245639
P6_1F	P6	*Formica*	*subaenescens*	Sagehen	Nevada	California	CW Torres	39.441583	-120.245639
P7_13P	P7	*Polyergus*	*mexicanus*	Sagehen	Nevada	California	CW Torres	39.441333	-120.244194
P7_14P	P7	*Polyergus*	*mexicanus*	Sagehen	Nevada	California	CW Torres	39.441333	-120.244194
P7_1F	P7	*Formica*	*argentea*	Sagehen	Nevada	California	CW Torres	39.441333	-120.244194
P8_15P	P8	*Polyergus*	*mexicanus*	Sagehen	Nevada	California	CW Torres	39.441444	-120.246333
P8_16P	P8	*Polyergus*	*mexicanus*	Sagehen	Nevada	California	CW Torres	39.441444	-120.246333
P8_1F	P8	*Formica*	argentea	Sagehen	Nevada	California	CW Torres	39.441444	-120.246333
P9_17P	P9	*Polyergus*	*mexicanus*	Sagehen	Nevada	California	CW Torres	39.435833	-120.261500
P9_1F	P9	*Formica*	*accreta* "B"	Sagehen	Nevada	California	CW Torres	39.435833	-120.261500
P9_18P	P9	*Polyergus*	*mexicanus*	Sagehen	Nevada	California	CW Torres	39.435833	-120.261500
Pol499	499	*Polyergus*	*mexicanus*	Sagehen	Nevada	California	JR Simms	39.436918	-120.242106
Pol537	537	*Polyergus*	*mexicanus*	Sagehen	Nevada	California	JR Simms	39.443494	-120.264795
Pol549	549	*Polyergus*	*mexicanus*	Sagehen	Nevada	California	SA Cooper	39.452363	-120.256382
Pol550	550	*Polyergus*	*mexicanus*	Sagehen	Nevada	California	JR Sapp	39.458085	-120.236496
Pol550B	550	*Polyergus*	*mexicanus*	Sagehen	Nevada	California	JR Sapp	39.458085	-120.236496
Pol551	551	*Polyergus*	*mexicanus*	Sagehen	Nevada	California	JR Sapp	39.434619	-120.2411
Pol764	764	*Polyergus*	*mexicanus*	Sagehen	Nevada	California	JR Sapp	39.432398	-120.243866
Pol782	782	*Polyergus*	*mexicanus*	Sagehen	Nevada	California	JR Sapp	39.454345	-120.255692
BCL_P	PSW15985	*Polyergus*	*mexicanus*	Blue Canyon Lake	Tuolumne	California	PS Ward	38.29833	-119.66525
BCL_F	PSW15986	*Formica*	*accreta* "A"	Blue Canyon Lake	Tuolumne	California	PS Ward	38.29833	-119.66525
