# Supplementary material for: Genetic and chemical divergence among host races of a socially parasitic ant
Source: Ecol Evol. 2018 Nov 6;8(23):11385–98. doi: 10.1002/ece3.4547 (PMC6303767; doi:10.1002/ece3.4547)
Supplement: Supplementary file 2 [file ECE3-8-11385-s002.pdf]

Appendix 2. Variations in 10µl PCR conditions for 8 microsatellite loci.

Locus	$MgCl_2$	Taq	T_a[1]
pol 1	1.25 mM	0.075 units	59.5˚C
pol 2	2 mM	0.075 units	58˚C
pol 3	2 mM	0.075 units	59.5˚C
pol 4	1.25 mM	0.075 units	59.5˚C
pol 5	2 mM	0.075 units	54.6˚C
pol 12	1.75 mM	0.075 units	58˚C
Fy 4, 13	1.25 mM	0.04 units	48˚C

[1] Annealing temperature
