# Supplementary material for: Genetic and chemical divergence among host races of a socially parasitic ant
Source: Ecol Evol. 2018 Nov 6;8(23):11385–98. doi: 10.1002/ece3.4547 (PMC6303767; doi:10.1002/ece3.4547)
Supplement: Supplementary file 3 [file ECE3-8-11385-s003.pdf]

Appendix 3. Chemical peaks accounting for >50% of the differences between *Polyergus* individuals grouped by host species. Question marks indicate likely, but uncertain, identities (in some cases, multiple compounds co-eluted at the same retention time). X indicates presence of the compound; blank indicates absence.

CHC compound(s)	Retention time (min)	*F. accreta* "A" vs. *F. argentea*	*F. subaenescens* vs. *F. accreta* "A"	*F. subaenescens* vs. *F. argentea*
C21	10.620	X		
9-MeC21; 11-MeC21	11.088	X		
C23	13.414	X	X	X
9- MeC23; 11-MeC23	13.980	X	X	
C24	16.897	X	X	X
11-MeC25; 13-MeC25	17.539	X	X	X
C26	20.777	X	X	X
13,16-diMeC26; 9,10-diMeC26?	21.451	X	X	
7,18-diMeC26?	21.566	X		
7,19,23-triMeC26?	22.233		X	X
5,9,11-triMeC26?	22.394			X
3,7-diMeC27; 13-MeC27	22.857		X	X
8,12-diMeC28?	23.499		X	X
C29	24.794			X
11-MeC29; 13-MeC29	25.403	X	X	X
9-MeC29	25.491	X	X	
11,15-diMeC29	25.911	X		
3,13-diMeC29	26.866		X	X
11,15-diMeC31	29.860	X		
7,x-diMeC31; 9,x-diMeC31	30.091	X		
11,21-diMeC33	33.809	X		
